# Bone mineral density assessment by DXA in rheumatic patients with end-stage osteoarthritis undergoing total joint arthroplasty

**DOI:** 10.1186/s12891-021-04039-5

**Published:** 2021-02-11

**Authors:** Moritz Mühlenfeld, André Strahl, Ulrich Bechler, Nico Maximilian Jandl, Jan Hubert, Tim Rolvien

**Affiliations:** 1 Department of Orthopedics, University Medical Center Hamburg-Eppendorf, Martinistrasse 52, 20246 Hamburg, Germany; 2 Department of Osteology and Biomechanics, University Medical Center Hamburg-Eppendorf, Lottestrasse 59, 22529 Hamburg, Germany

**Keywords:** Rheumatoid arthritis, Arthroplasty, DXA, Bone mineral density

## Abstract

**Background:**

Patients with rheumatic diseases have a high risk for joint destruction and secondary osteoarthritis (OA) as well as low bone mineral density (BMD, i.e., osteoporosis). While several factors may lead to low BMD in these patients, the value of BMD measurements in rheumatic patients with end-stage OA scheduled for total joint arthroplasty is unknown.

**Methods:**

In this retrospective cross-sectional study of 50 adults with secondary OA due to rheumatic diseases, we evaluated dual energy X-ray absorptiometry (DXA) measurements of both hips and the spine performed within 3 months prior to arthroplasty (*n* = 25 total hip arthroplasty, THA; *n* = 25 total knee arthroplasty, TKA). We analyzed various demographic and disease-specific characteristics and their effect on DXA results by using group comparisons and multivariate linear regression models.

**Results:**

Although patients undergoing TKA were younger (63.2 ± 14.2 vs. 71.0 ± 10.8 yr., *p* = 0.035), osteoporosis was observed more frequently in patients scheduled for TKA than THA (32% vs. 12%). Osteopenia was detected in 13/25 patients (52%) in both the THA and TKA cohort. In the THA cohort, female sex, lower BMI and prednisolone use were associated with lower T-score in the hip. In TKA patients, higher OA grade determined by Kellgren-Lawrence score was associated with lower T-score in the hip of the affected side.

**Conclusions:**

Osteoporosis is present in a considerable frequency of rheumatic patients with end-stage OA, and THA and TKA patients show distinct frequencies and risk factors of low BMD. Our findings point to a potential value of DXA regarding preoperative evaluation of bone status.

## Background

Patients with rheumatoid arthritis (RA) and other rheumatic diseases have a high risk for joint destruction and secondary osteoarthritis (OA) as well as systemic bone loss reflected by low bone mineral density (BMD, i.e., osteoporosis) [1, 2]. Total joint arthroplasty (TJA) represents a highly successful surgical treatment option to restore the joint function of the hip and knee joint in RA patients [3–5]. Due to the presence of various risk factors such as chronic inflammation or corticosteroid use, the risk of osteoporosis was found to be 2-fold higher in RA patients compared to the general population [2]. Systemic bone loss in RA and other rheumatic diseases is mediated through an altered balance between bone formation and bone resorption. Specifically, pro-inflammatory cytokines may promote both increased bone resorption and decreased bone formation [6].

While it was previously reported that osteoporosis is common and undertreated in primary OA patients who were screened prior to arthroplasty [7], the risk of osteoporosis in RA patients with end-stage OA undergoing arthroplasty may be even higher. Furthermore, it remains unclear whether BMD assessed by dual energy X-ray absorptiometry (DXA) may facilitate the decision to perform either cemented or cementless TJA. This decision is usually influenced by age, bone configuration (i.e., bone geometry, Dorr classification in plain radiographs) as well as intraoperative impression of bone quality. Although certain patient groups may be more suitable for one or another, the survivorship of cemented vs. cementless hip and knee arthroplasties was reported to be overall similar in primary OA patients [8, 9]. In RA patients undergoing total hip arthroplasty (THA), cementless femoral components are associated with good clinical outcomes, although a number of bone-related complications including osteolysis (30%) and subsidence (14%) [4] as well as periprosthetic fracture (5.2%) [10] were previously reported. Good to excellent outcomes were also reported for both cemented and cementless total knee arthroplasty (TKA) in RA patients [5, 11, 12]. Nevertheless, the overall associations between bone health (particularly BMD) and the decision for cemented or cementless TJA are not known [13].

In this study, we evaluated the BMD values that were assessed preoperatively by DXA in 50 patients with rheumatic diseases undergoing total joint (hip and knee) arthroplasty. We hypothesized that osteoporosis is common and undertreated in these patients.

## Methods

### Study cohort

We retrospectively reviewed 50 consecutive patients with advanced joint destruction and/or end-stage secondary OA due to rheumatic diseases who underwent TJA in our clinic between 2018 and 2019. All surgical procedures were performed by senior orthopedic surgeons with > 5 years of experience in the field of joint replacement surgery at our department. Demographic and disease-specific characteristics including age, sex, BMI, DXA results, glucocorticoid and methotrexate (MTX) use as well as the type of prosthesis fixation (cemented vs. cementless) were assessed. The diagnosis of the respective rheumatic disease had been made externally according to the official criteria (e.g., ACR/EULAR). The radiological severity of OA was determined by the Kellgren-Lawrence score [14]. In the THA group, all acetabular cups were implanted cementless while cementation of the femoral stem (“hybrid”) varied among the cases. In the TKA group, cementation of the femoral component varied between cemented or cementless, while tibial fixation was cemented in all cases. The decision to perform either cemented or cementless fixation of the femoral component in THA and TKA depended, among other factors, on age, sex, BMI and femur geometry. DXA measurements were not included in this decision in a routine or standardized manner. This retrospective study was performed according to the rules of the local ethics committee. A retrospective chart analysis was conducted, and data were anonymized before further analysis.

### DXA

Due to the presence of rheumatic diseases, DXA was performed in our radiological department in all patients to assess fracture risk and determine the indication for osteoporosis therapy according to the guidelines of the German Society for Osteology (DVO). BMD was measured by DXA (Lunar Prodigy enCore 2007, GE Healthcare; Madison, WI, USA). Only patients with available DXA measurements within 3 months prior to surgery were included. Specifically, both proximal femur and the lumbar spine (L1-L4) were evaluated by DXA. From these results, we determined the T- and Z-scores, which represent the standard deviation compared to 20 to 40-year-old, sex-matched healthy controls and age−/sex-matched controls, respectively. The diagnosis of osteoporosis and osteopenia was determined according to the classification of the world health organization (WHO, osteoporosis T-score ≤ − 2.5, osteopenia T-score ≤ − 1.0). In the THA cohort, DXA could not be performed in the contralateral proximal femur due to previous THA in 6/25 patients. In the TKA cohort, DXA could not be performed in the ipsilateral and contralateral femur due to previous THA in 3/25 and 1/25 patients, respectively. DXA could not be performed in the lumbar spine due to previous lumbar fusion in 1/25 THA patients.

### Statistics

SPSS statistical program 25.0 (SPSS, Chicago, IL) and GraphPad Prism 7 (GraphPad Software, La Jolla, CA) were used for statistical analyses. Continuous variables are expressed as mean ± standard deviation (SD), while categorial variables are expressed as number and percentage. Normality of data was tested using the D’Agostino & Pearson test. For correlation analyses, Pearson’s correlation test was used for normally distributed data, and Spearman’s rank correlation test was used for non-normally distributed data. To test for differences between the study groups, we used the unpaired *t*-test for normally distributed data and the Mann–Whitney U test for non-normally distributed data. When comparing more than two groups, significance was calculated by the Kruskal-Wallis test with Dunn’s multiple comparisons test. Finally, to determine possible predictors of BMD in the THA and TKA cohorts, we performed a multivariate regression model with age, sex, body mass index (BMI), Kellgren-Lawrence score and prednisolone dose as predictors of the T-score of the operated and non-operated side as well as the lumbar spine including all predictors at a single step. *P*-values of < 0.05 were considered as statistically significant.

## Results

### Demographic and disease-specific characteristics of the study population

From a total of *n* = 50 patients, we analyzed 25 patients with THA and 25 patients with TKA. An overview of the demographic and disease-specific characteristics of the study population can be found in Table 1. TKA patients were significantly younger on average (*p* = 0.035), and the percentage of female patients was higher (*p* = 0.045). Both groups were similar in age, height, BMI and number of smokers (*p* > 0.05). A major part of both groups consisted of patients with RA (84% for both groups) with only few patients with psoriatic arthritis (PsA) or ankylosing spondylitis (AS). While a trend towards more patients receiving glucocorticoids was observed in the TKA group (*p* = 0.152), the mean glucocorticoid dose was similar in both groups. The same applied for the percentage and dosage of methotrexate (MTX). Cemented fixation of the femoral stem (“hybrid”) was performed in 44% of the THA patients. In the TKA group, cemented fixation of the femoral component was performed in 56%. The radiological severity of OA determined by Kellgren-Lawrence score was similarly high in both the THA and TKA patients (*p* = 0.837).

**Table 1:** Demographic, disease-specific, and DXA characteristics of rheumatic patients scheduled for THA and TKA

	THA	TKA	
	Mean (SD) or n (%)	Mean (SD) or n (%)	*p*-value
**Demographic characteristics**			
Females (%)	11/25 (44%)	18/25 (72%)	0.045*^#^
Age (yr.)	71.0 (10.8)	63.2 (14.2)	0.035*
Height (cm)	170.2 (8.2)	166.6 (10.6)	0.093
Weight (kg)	77.4 (13.4)	78.1 (16.2)	0.861
BMI (kg/m^2^)	26.6 (3.6)	28.5 (5.4)	0.150
Smoking (%)	4/25 (16%)	4/25 (16%)	1.0^#^
**Disease & treatment regimen**			
RA	21/25 (84%)	21/25 (84%)	1.000^#^
PsA	1/25 (4%)	3/25 (12%)	0.297^#^
AS	3/25 (12%)	1/25 (4%)	0.297^#^
Glucocorticoids	12/25 (48%)	17/25 (68%)	0.152
Glucocorticoid dose (mg)	5.3 (2.7)	5.4 (3.3)	0.937
MTX (%)	12/25 (48%)	17/25 (68%)	0.152^#^
MTX dose (mg)	14.2 (2.9)	14.7 (4.0)	0.719
Biologicals (%)	6/25 (24%)	4/25 (16%)	0.480^#^
**OA severity & implant fixation**			
Kellgren-Lawrence Score	3.1 (0.73)	3.1 (0.64)	0.837
Cemented femoral component fixation	11/25 (44%)	14/25 (56%)	0.396^#^
**DXA & osteopenia/osteoporosis**			
T-score – femur lowest	−1.0 (1.3)	−1.7 (1.2)	0.067
Z-score – femur lowest	0.0 (1.2)	−0.9 (1.2)	0.010^*^
T-score – affected femur	−0.8 (1.3)	−1.4 (1.3)	0.114
Z-score – affected femur	0.2 (1.2)	−0.7 (1.2)	0.013^*^
T-score – contralateral femur	−0.8 (1.1)	−1.4 (1.2)	0.136
Z-score – contralateral femur	0.3 (0.9)	−0.6 (1.2)	0.012^*^
T-score – lumbar spine	−0.5 (2.1)	−1.1 (1.8)	0.333
Z-score – lumbar spine	0.4 (1.8)	−0.4 (2.2)	0.180
Osteoporosis (T-score ≤ −2.5)	3/25 (12%)	8/25 (32%)	0.088^#^
Osteopenia (T-score ≤ −1 - ≤ −2.5)	13/25 (52%)	13/25 (52%)	1.000^#^
Vitamin D supplementation	6/25 (24%)	7/25 (26%)	0.747^#^
Bone-specific treatment	1/25 (4%)	2/25 (8%)	0.552^#^
Indication bone-specific treatment	10/25 (40%)	11/25 (44%)	0.774^#^

^*^indicates significant differences. ^#^determined by the Chi^2^ test

### Characterization of bone mineral density in rheumatic THA and TKA patients

**Fig. 1:**
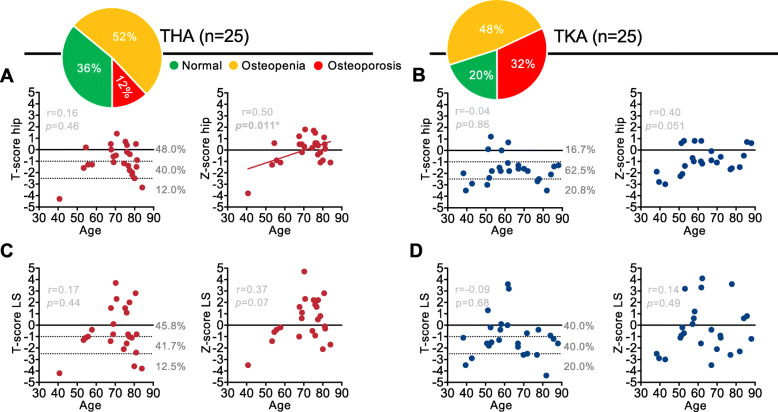
Overview of DXA values in THA/TKA patients with age, and diagnosis of osteoporosis/osteopenia based on T-scores. Pie charts indicate the frequency of osteoporosis, osteopenia and normal BMD in the TKA and the THA cohort. **a** Individual T- and Z-scores in the hip in the THA cohort with age. **b** Individual T- and Z-scores in the hip in the TKA cohort. **c** Individual T- and Z-scores in the lumbar spine (LS) in the THA cohort. **d** Individual T- and Z-scores in the lumbar spine (LS) in the TKA cohort. Linear correlation analysis for all panels, *p < 0.05

To characterize the overall skeletal status in the rheumatic patients with THA and TKA, we first determined the BMD and respective T- and Z-scores by DXA of the total hip and the lumbar spine (Fig. 1a-d, Table 1). Among the 25 THA patients analyzed, three individuals (12%) were diagnosed with osteoporosis (T-score ≤ − 2.5), whereas ten individuals (40%) displayed osteopenia (T-score ≤ − 1.0) in the hip (Fig. 1a). The Z-score in the hip correlated positively with age (*p* = 0.011), while no correlation between age and T-score was observed. In the lumbar spine, similar DXA results were observed, with 3/24 individuals (12.5%) being in the range of osteoporosis and 10/24 individuals (41.7%) displaying osteopenia (Fig. 1c). Age had no significant influence on T- or Z-score. Since the same three patients had osteoporotic T-scores in the hip and lumbar spine, three of the 25 patients in total (12%) were diagnosed with osteoporosis, while 13/25 (52%) were diagnosed with osteopenia according to the WHO guidelines. Of the osteoporotic patients, 2/3 had not been previously diagnosed with osteoporosis or received adequate treatment.

In the analyzed TKA cohort, we found a higher frequency of low bone mass, although these patients were significantly younger than the patients undergoing THA. Namely, 5/24 individuals (20.8%) were diagnosed with osteoporosis (T-score ≤ − 2.5), whereas 15 individuals (62.5%) displayed osteopenia (T-score ≤ − 1.0) in the hip (Fig. 1 B). In the lumbar spine, 5/25 individuals (20%) were in the range of osteoporosis and ten individuals (40.0%) displayed osteopenia (Fig. 1d). No age effects could be detected. Considering the lowest T-score of any measurement site according to the WHO guidelines, eight patients in total (32%) were diagnosed with osteoporosis, and 13/25 (52%) were diagnosed with osteopenia. Six of these eight osteoporotic patients had not been previously diagnosed with osteoporosis or received adequate treatment.

The frequency of vitamin D supplementation was 6/25 (24%) and 7/25 (28%) for THA and TKA, respectively (Table 1). While there was an indication for pharmacologic, bone-specific treatment in 10/25 THA and 11/25 TKA patients (based on the guidelines of the German Society for Osteology that includes DXA results and risk factors), only 1/25 of the THA and 2/25 of the TKA patients received such treatment (Table 1).

**Fig. 2:**
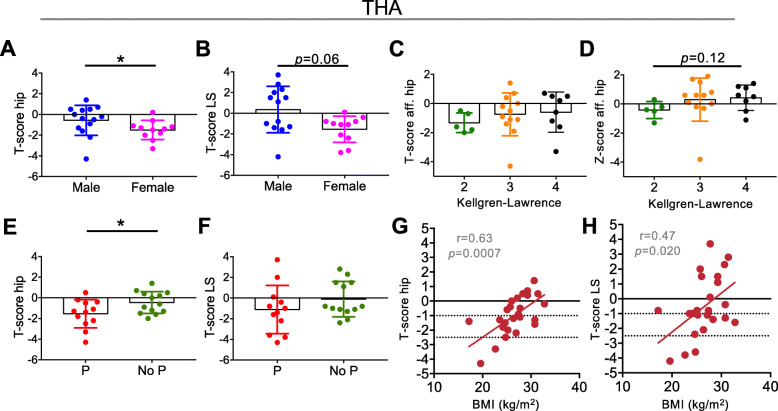
Characterization of contributing factors to DXA values in THA patients. **a**, **b** Comparison of T-scores in the hip (lowest of both sides) and the lumbar spine (LS) between male and female THA patients. **c**, **d** T-scores and Z-scores in the affected hip in relation to Kellgren-Lawrence score subgroups. **e**, **f** Comparison of T-scores in the hip (lowest) and LS between THA patients with current prednisolone use (P) and no prednisolone therapy (No P). **g**, **h** Correlation analysis between body mass index (BMI) and T-score in the hip and LS in THA patients. *p < 0.05, **p < 0.001

We next evaluated the contributing factors to DXA results in both THA and TKA patients. In THA patients, female sex was associated with significantly lower T-score in the hip (lowest of both sides) and a trend towards lower T-score in the lumbar spine (Fig. 2a, b). The radiological severity of OA (i.e., Kellgren-Lawrence score), which includes the severity of osteophyte formation and subchondral sclerosis, was not associated with differences in the T- or Z- scores and the hip (Fig. 2c, d). Current prednisolone use was associated with significantly lower T-score in the hip (lowest) and had no significant effect on DXA T-scores in the spine (Fig. 2e, f). BMI correlated positively with T-score in the hip and the lumbar spine (Fig. 2g, h). Smoking showed no significant influence on T-score in the hip or the lumbar spine (*p* > 0.05).

**Fig. 3:**
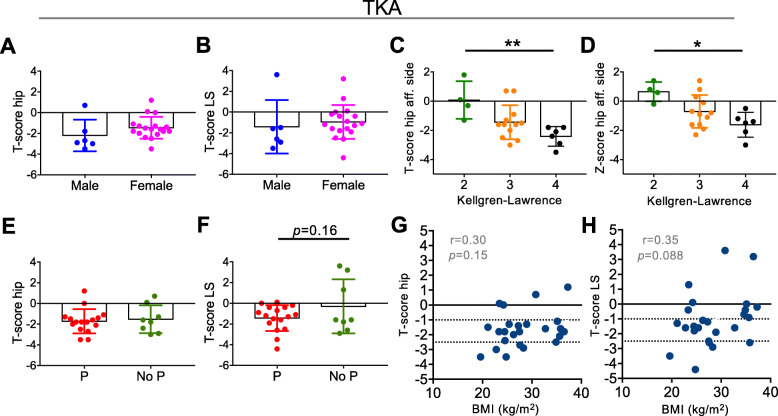
Characterization of contributing factors to DXA values in TKA patients. **a**, **b** Comparison of T-scores in the hip (lowest) and lumbar spine (LS) between male and female TKA patients. **c**, **d** T-scores and Z-scores in the affected (unilateral) hip in relation to Kellgren-Lawrence score subgroups. **e**, **f** Comparison of T-scores (lowest) in the hip and LS between TKA patients with current prednisolone use (P) and no prednisolone therapy (No P). **g**, **h** Correlation analysis between body mass index (BMI) and T-score in the hip and LS in TKA patients. *p < 0.05, **p < 0.001

**Fig. 4:**
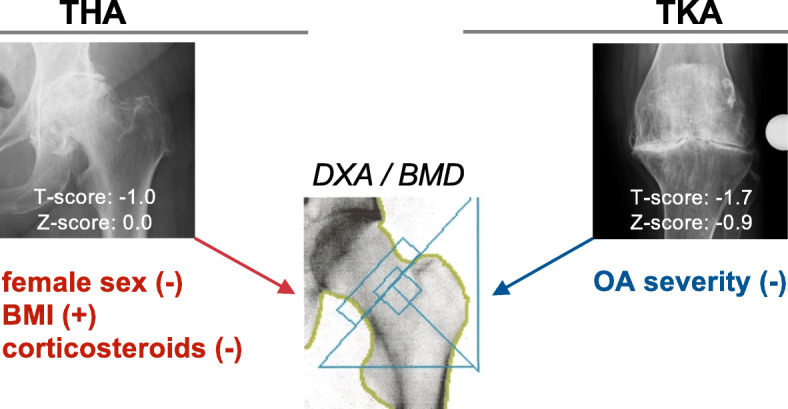
Schematic explanation for the detected differences in DXA values between the THA and TKA cohort. (+) factors with positive influence on BMD, (−) factors with negative influence on BMD

In the TKA patients, sex had no influence on T-score (Fig. 3a, b). BMD expressed by T- and Z-score was significantly lower in patients with high OA grade (i.e., Kellgren-Lawrence score of 4) compared to low OA grade (i.e., Kellgren-Lawrence score 2) (Fig. 3c, d). Current prednisolone treatment, BMI and smoking had no significant influence on T-score in the hip or lumbar spine, although a trend towards lower T-score in the lumbar spine with prednisolone treatment could be observed (Fig. 3e-h). The contributing factors to BMD values in the THA and the TKA cohort are visualized in Fig. 4 (Fig. 4).

### Predictors for bone mineral density in in rheumatic patients undergoing THA or TKA

A multiple linear regression was carried out to identify independent predictors for BMD represented by the T-score of the operated and non-operated side as well as the lumbar spine. The results of THA patients demonstrated that BMI could significantly predict the patients’ T-score in the affected hip (β = 0.667, *p* = 0.001) but not in the lumbar spine. The regression model significantly explained 44.6% of the variance (F _(5, 17)_ = 4.54, *p* = 0.008). On the contralateral non-operated side, BMI (β = 0.665, *p* = 0.005) and prednisolone dose (β = − 0.439, *p* = 0.028) served as independent predictors for BMD with an explained variance of 58.5% (F _(5, 11)_ = 5.51, *p* = 0.009). In the analyzed TKA cohort, only the Kellgren-Lawrence score showed a significant influence on the T-score at the operated side (β = − 0.597, *p* = 0.008). However, the overall significant explained variance of 34.2% (F _(5, 14)_ = 2.98, *p* = 0.049) was smaller than in THA patients cohort. No significant predictor could be identified for BMD in the lumbar spine (Table 2).

**Table 2:** Predictors for DXA parameters in the THA and TKA cohort using a multivariate regression model

	Operated side			Contralateral non-operated side			Lumbar spine		
	β	T	*p*	β	T	*p*	β	T	*p*
**THA patients**									
(Intercept)		- 4.226	0.001		- 2.243	0.046		- 2.415	0.028
Age	0.017	0.100	0.922	- 0.262	- 1.542	0.151	0.194	0.948	0.357
Sex	0.132	0.764	0.455	0.170	0.984	0.346	0.412	1.969	0.066
BMI	**0.667**	**3.807**	**0.001**	**0.665**	**3.459**	**0.005**	0.385	1.857	0.082
KL score	0.253	1.489	0.155	0.176	1.014	0.332	- 0.032	- 0.162	0.873
Prednisolone dose	- 0.177	- 1.974	0.298	**- 0.439**	**- 2.538**	**0.028**	0.088	0.440	0.666
R^2^ adjusted	0.446**			0.585**			0.265		
**TKA patients**									
(Intercept)		0.954	0.356		0.289	0.776		- 0.112	0.912
Age	- 0.266	- 1.342	0.201	- 0.179	- 0.844	0.411	- 0.259	- 1.176	0.256
Sex	- 0.126	- 0.606	0.554	- 0.237	- 1.097	0.289	- 0.075	- 0.347	0.733
BMI	0.252	1.284	0.220	0.306	1.478	0.159	0.421	1.955	0.067
KL score	**- 0.597**	**- 3.093**	**0.008**	**- 0.467**	**- 2.351**	**0.032**	- 0.250	- 1.196	0.248
Prednisolone dose	- 0.011	- 0.051	0.960	- 0.213	- 1.062	0.304	- 0.311	- 1.488	0.155
R^2^ adjusted	0.342*			0.223			0.098		

Bold indicates significant independent predictors (*p* < 0.05). Asterisks indicate significance in the overall ANOVA multiple regression prediction model (*p < 0.05,**p < 0.01). *BMI* Body mass index, *KL* Kellgren-Lawrence

## Discussion

TJA represents a widely performed surgical procedure to restore the function of an osteoarthritic joint and to reduce pain. Given the fact that rheumatic patients have an increased risk for impaired bone quality, bone health assessment seems to be a logical preoperative measure. In the present study, we demonstrated the frequency of osteoporosis and osteopenia in a collective of rheumatic patients with end-stage OA. While a considerable frequency of osteoporosis was detected for patients scheduled for THA and TKA, a much higher proportion of patients had osteopenia, which is also known to be associated with increased fracture risk. Our data also underlined the relevant undertreatment in terms of bone-specific (anti-osteoporotic) medication.

Specifically, we evaluated the BMD of THA compared to TKA patients and depending on factors such as age, OA severity and current prednisolone treatment. While the frequency of osteoporosis was markedly higher in TKA compared to THA (32% vs. 12%), age was not associated with T-score in either the THA or TKA cohort. However, we found a strong positive association between BMI and T-score in patients with end-stage hip OA undergoing THA. In the TKA cohort, a negative association between knee OA grade (i.e., Kellgren-Lawrence score) and T-score in the unilateral hip was detected. Indeed, although TKA patients were markedly younger, this cohort consisted of more corticosteroid users and more female patients, which may explain the higher frequency of osteoporosis. While it is known the corticosteroids lead to low bone mass in RA [2], current prednisolone treatment was associated with lower BMD values in the hip in THA but not TKA patients. In this regard, the lack of significant differences in the TKA cohort could be due to the fact that most of the patients without current prednisolone treatment had been previously treated with corticosteroids. In multiple regression models, we additionally identified prednisolone treatment as a negative predictor of BMD in the non-operated contralateral hip.

A reason for higher BMD values in the THA group might be due to local degenerative changes such as osteophytes that positively influence DXA results, since the measurement site of hip DXA corresponds to the region in which secondary hip OA is observed. This hypothesis is supported by the observation that BMD values showed an increasing trend with the progression of OA severity and age in THA patients, while DXA values were significantly lower with higher Kellgren-Lawrence scores in the TKA patients. In general, it is well known that the radiological manifestations of OA (primarily osteophytes) positively influence BMD, which may lead to false-high measurement results in the THA cohort. While this phenomenon has been primarily reported for the lumbar spine [15], false-high DXA results due to local advanced joint destruction indicate the need for high-resolution peripheral procedures such as high-resolution peripheral quantitative computed tomography (HR-pQCT), which have been shown to predict fragility fractures independently of DXA values [16, 17]. The finding that radiologically more severe OA was associated with lower BMD in the TKA cohort is compatible with another previous study, in which an association between high radiological RA damage and low BMD in the hip was detected [18]. Interestingly, there are also examples for an inverse relationship between low BMD (i.e., osteoporosis) and OA, one of them being the observation that individuals with high bone mass have an increased prevalence of knee OA [19].

Although cemented and cementless fixation techniques in THA and TKA were reported to have overall comparable clinical outcomes [5, 20, 21], objective preoperative indicators to facilitate the decision on cemented or cementless fixation are lacking. In this context, subtle differences between the two fixation methods have been outlined previously (primarily for THA). In patients undergoing THA, it has been demonstrated that cementless stems have a higher risk of periprosthetic (intraoperative) femur fractures and stem subsidence compared to cemented stems [22–25]. Since RA patients have a higher risk for both low BMD and periprosthetic fractures [26], it seems reasonable that the selection of the most appropriate fixation procedure (i.e., cemented vs. cementless) should be made on the basis of the BMD. In other words, DXA may be beneficial to identify patients with a high risk for periprosthetic fracture, and specific thresholds or cutoff values could facilitate the decision to favor cemented over cementless implant fixation. As an overall 2.5-fold increase of periprosthetic fracture incidence has been observed in the past two decades [27], clinical efforts should aim to minimize periprosthetic fracture rates. Risk stratification could also rely on DXA measurements in the future as it is the case for other osteoporotic fractures. For cemented stems in THA, previous studies reported that revision rates > 10 years were even lower in RA compared to OA patients, while no differences in revision rates were found for TKA in RA vs. OA patients [28, 29]. In this regard, it is also interesting to note that the survivorship of cementless stems in THA was reported to be negatively influenced by corticosteroid treatment [10]. In patients undergoing TKA, cementless techniques are increasingly used. Early studies in RA patients with cemented procedures show overall comparable success rates compared to more recent studies with cementless procedures [5, 21, 30, 31]. Given these collective findings, it is likely that THA patients could benefit from DXA measurement prior to surgery and DXA could be used for the appropriate planning of the surgery. While the retrospective data of the present study did not allow us to determine a specific DXA threshold below which cemented fixation should be favored over cementless fixation, further longitudinal and randomized-controlled studies should evaluate the values of DXA results regarding the survivorship and complication rates of cemented vs. cementless fixation techniques.

Our study has a few limitations. Due to the low number of cases, the results may only show general trends and should be interpreted with caution. Further studies with larger sample sizes should be targeted in the future. Furthermore, it was not possible to fully distinguish whether the joint destruction had come from the rheumatic disease, OA or both from the plain radiographs, although we observed the hallmark features of OA such as osteophytes. It is commonly accepted that the primary radiological feature of RA is bone erosion, which, due to repair mechanisms, can lead to secondary OA, which is primarily characterized by proliferative changes such as osteophytes [32]. We are also aware of the fact that our study does not provide sufficient evidence regarding the clinical outcome of the analyzed patients. Future longitudinal studies should evaluate the influence of BMD on the clinical outcome and complications of cemented vs. cementless implant fixation at long-term follow-up.

## Conclusion

DXA represents a noninvasive measurement to determine the BMD in patients with rheumatic diseases prior to arthroplasty. Patients undergoing TKA suffered more frequently from osteoporosis compared to THA (32% vs. 12%), possibly due to local proliferative changes, which are also captured in the DXA measurements of the hip. Whether BMD values influence the survivorship and complication rates in cemented vs. cementless fixations in patients with rheumatic diseases remains to be established in studies with longitudinal designs.

## Data Availability

The datasets used and/or analyzed during the current study are available from the corresponding author on reasonable request.
